# Dynamic SARS-CoV-2-specific B-cell and T-cell responses induced in people living with HIV after a full course of inactivated SARS-CoV-2 vaccine

**DOI:** 10.3389/fimmu.2025.1554409

**Published:** 2025-02-25

**Authors:** Xiuwen Wang, Xiaodong Yang, Xin Zhang, Hongxia Yan, Junyan Jin, Zhenglai Ma, Junyi Duan, Guanghui Zhang, Tao Huang, Yongzheng Li, Hao Wu, Tong Zhang, Aiwei Zhu, Cong Jin, Xiangrong Song, Bin Su

**Affiliations:** ^1^ Beijing Key Laboratory for HIV/AIDS Research, Sino-French Joint Laboratory for HIV/AIDS Research, Clinical and Research Center for Infectious Diseases, Beijing Youan Hospital, Capital Medical University, Beijing, China; ^2^ Center for General Practice Medicine, Department of Rheumatology and Immunology, Zhejiang Provincial People’s Hospital (Affiliated People’s Hospital), Hangzhou Medical College, Hangzhou, Zhejiang, China; ^3^ Department of Infectious Diseases, The First Affiliated Hospital of Xi’an Jiaotong University, Xi’an, Shaanxi, China; ^4^ Tian Yuan Studio, Beijing Youan Hospital, Capital Medical University, Beijing, China; ^5^ Department of Pathogeny Biology, College of Basic Medical Sciences, Jilin University, Changchun, Jilin, China; ^6^ National Center for AIDS/STD Control and Prevention, Chinese Center for Disease Control and Prevention, Beijing, China; ^7^ Department of Critical Care Medicine, Frontiers Science Center for Disease related Molecular Network, State Key Laboratory of Biotherapy and Cancer Center, West China Hospital, Sichuan University, Chengdu, Sichuan, China; ^8^ Central Laboratory, Beijing Youan Hospital, Capital Medical University, Beijing, China

**Keywords:** HIV, SARS-CoV-2 vaccine, B cells, T cells, immune responses

## Abstract

**Objective:**

Both B-cell- and T-cell-mediated immunity are crucial for the effective clearance of viral infection, but little is known about the dynamic characteristics of SARS-CoV-2-specific B-cell and T-cell responses in people living with HIV (PLWH) after a full course of inactivated SARS-CoV-2 vaccination.

**Methods:**

In this study, fifty people living with HIV (PLWH) and thirty healthy controls (HCs) were enrolled to assess B-cell and T-cell responses at the day before the vaccination (T0), two weeks after the first dose (T1), two months after the first dose (T2), the day of the third dose (T3), one month after the third dose (T4), three months after the third dose (T5) and 12 months (T6) after the third dose.

**Results:**

SARS-CoV-2-specific B-cell and T-cell responses were induced in people living with HIV (PLWH), and these responses lasted at least one year after the third vaccine dose. However, the peak frequencies of Spike-specific B-cell and T-cell responses in PLWH were lower than those in HIV-negative controls. In addition, the expansion of activated B cells, memory B cells and plasma cells after primary vaccination was observed, but the percentages of these cells were decreased at T6 and were comparable to those at T0. Additionally, the percentages of activated T cells, exhausted T cells and SARS-CoV-2-specific T cells with enhanced functional activity were increased following the administration of inactivated SARS-CoV-2 vaccine. In addition, PLWH had lower percentages of plasma cells, RBD-specific B cells, circulating Tfh (cTfh) cells and CD38^+^ cTfh cells, and the percentages of the latter two types of cells were positively correlated with the titer of neutralizing antibodies, indicating these differences may account for the weaker immune responses induced in PLWH.

**Conclusion:**

These data suggest that specific B-cell and T-cell responses could be sustained for at least one year after receiving the third vaccination. Our findings emphasize that the weak SARS-CoV-2-specific B-cell and T-cell responses induced in PLWH have implications for clinical decision-making and public health policy for PLWH with respect to SARS-CoV-2 infection.

## Introduction

Accumulating evidence suggests that a broad and well-coordinated immune response is required for protection against SARS-CoV-2 infection. The emergence of variants of concern (VOCs) with increased ability to evade neutralizing antibodies (NAbs) has reinforced the need for a more comprehensive assessment of adaptive immunity after vaccination (1), especially in more vulnerable groups, including some people living with HIV (PLWH). Owing to compromised immunity, SARS-CoV-2 infection is considered a challenge for PLWH, who appear to have a greater risk of hospitalization and worse clinical outcomes, and this phenomenon is likely to be exacerbated in those with low CD4^+^ T-cell counts (2, 3). Previous data have shown that SARS-CoV-2 vaccination is safe and effective in most PLWH, but weaker antibody responses were observed following SARS-CoV-2 vaccination, especially in those with low CD4^+^ T-cell counts of less than 200 cells/μL (4, 5). In addition, breakthrough infections were found to occur and vaccine effectiveness was demonstrated to wane more rapidly in PLWH, who have weaker humoral and cellular immune responses after vaccination (6).

The immune response declines sharply at 6 months post-vaccination; hence, the administration of a booster dose has emerged as a potential strategy to enhance and sustain protective immune responses (7). In concert with robust humoral immunity, the preferential induction of antigen-specific B cells, follicular helper T (Tfh) cells, and CD4^+^ and CD8^+^ T cells indicates that the vaccine-elicited immune response is focused specifically on generating lasting protection against SARS-CoV-2 infection (8, 9). However, at six months post-vaccination, the antibody response in almost all individuals was found to drop below the detection limit, and the production of antigen-specific memory B cells (MBCs, marked as CD27^+^ B cells) and T cells was induced by booster vaccination and lasted for at least 6 months (10). Several reports have noted that the frequencies of MBCs with the ability to produce neutralizing antibodies are maintained or increase although the neutralizing antibody titer might decrease over the period between 5 and 12 months after SARS-CoV-2 infection or vaccination (11–15). Various studies focusing on SARS-CoV and MERS-CoV infection have also shown the importance of T-cell-mediated immunity in maintaining long-term immune protection and reducing clinical disease severity (16, 17). Several studies have shown that the frequencies of SARS-CoV-2-specific CD4^+^ and CD8^+^ memory T cells peak early, within the first month, but then slowly decline over the next 6–7 months in convalescent patients infected with SARS-CoV-2 (18). In addition, a study focused on the impact of SARS-CoV-2 variants on immune responses revealed that the titers of neutralizing antibodies against Omicron declined dramatically three months after the third dose, whereas memory CD4^+^ T-cell responses were maintained for at least eight months after the second dose and three months after the third mRNA vaccine dose (19). In addition, in chronic lymphocytic leukemia (CLL) and myeloid dysplastic syndrome (MDS) patients with impaired humoral responses, mRNA vaccines have the potential to elicit long-lasting CD8^+^ T-cell-mediated immunity (20). These findings highlight the importance of SARS-CoV-2-specific B and T cells in the long-term defense against SARS-CoV-2.

In PLWH, the humoral response after the booster dose is strongly enhanced (21, 22), whereas the titer of neutralizing antibodies produced in response to the third dose of the SARS-CoV-2 vaccine was lower in severely immunosuppressed HIV-infected individuals than in those with a CD4^+^ T-cell count of more than 350 cells/μL (23). As reported in previous studies, specific T-cell responses can be induced by SARS-CoV-2 vaccines, and the administration of additional shots has implications for enhancing T-cell responses in PLWH (24). However, HIV infection may lead to impaired T-cell responses, since there were fewer IFN-γ-secreting T cells in PLWH after receiving primary or booster vaccines (25, 26).

However, data on vaccine immunogenicity and durability in PLWH after a full course of inactivated SARS-CoV-2 vaccine remain limited. In addition, the role of HIV-associated immune dysfunction in serological and cellular outcomes after SARS-CoV-2 vaccination remains unclear. In this study, we conducted a prospective, observational cohort study in a tertiary hospital in Beijing, China. We aimed to evaluate the dynamic characteristics of B- and T-cell responses in PLWH following the primary and booster SARS-CoV-2 vaccine doses.

## Materials and methods

### Study cohort and sample collection

A total of 50 PLWH who had received antiretroviral therapy (ART) for more than two years (with undetectable viral load and CD4 T cells above 350 cells/μL) and 30 healthy controls (HCs) were enrolled in this study at Beijing Youan Hospital. In addition, we excluded patients with major organ dysfunction, organ transplant recipients, with malignant tumors or undergoing chemotherapy, or who use immunosuppressants. The study was approved by Beijing Youan Hospital Research Ethics Committee (Nos. 2021-031 and 2021- 079). All participants tested negative for SARS-CoV-2 infection at the time of screening and provided written informed consent. The clinical trial protocol was approved by the hospital ethics committee. All the subjects received inactivated vaccines (CoronaVac). Blood samples were collected at several time points as mentioned above. Heparinized tubes were used for the collection of PBMCs and plasma via density gradient centrifugation. Blood was decanted into Leucosep tubes (Greiner Bio-One) containing Lymphoprep (STEMCELL Technologies) and centrifuged at 1,000 × g for 15 min with the brake off. The PBMCs were preserved in liquid nitrogen until the last follow-up was completed.

### Peptides and stimulation

Peptides covering the full-length SARS-CoV-2 wild-type and Omicron spike protein sequences were synthesized for use in antigen-specific T-cell assays (ProImmune). A total of 253 peptides were synthesized as 15-mers overlapping by ten amino acids. The two peptide pools used in our study were created by pooling aliquots of individual peptides and resuspending them in RPMI 1640 medium and DMSO at a concentration of 50 μg/mL. The peptide pools were used at a final concentration of 0.25 μg/mL, and an equimolar DMSO concentration was used in the nonstimulated control groups.

### *Ex vivo* IFN-γ ELISpot assays

IFN-γ ELISpot assays were performed according to the manufacturer’s instructions (Mabtech). First, plates were washed with sterile phosphate-buffered saline (PBS) and blocked with RPMI 1640 medium containing 10% FBS (Bovogen) for 20 minutes at room temperature (RT). A total of 2x10^5^ PBMCs per well were seeded and stimulated with the SARS-CoV-2 Spike pool (0.25 μg/mL) for 24 hours at 37°C. An anti-CD3 antibody (0.25 μg/mL) was used as a positive control and an equimolar concentration of DMSO as a negative control. The plates were washed with PBS and incubated with a biotinylated human monoclonal anti-IFN-γ antibody (0.25 μg/mL) for 2.5 hours at room temperature. After the samples were washed 3 times with PBS, streptavidin–alkaline phosphatase (Invitrogen) was added. The precipitates were detected with AP color reagent (Bio-Rad), and the reaction was stopped by rinsing with distilled water. Spot-forming units were quantified with an automated ELISpot reader (AID). To quantify antigen-specific responses, the mean spot intensities in the DMSO control wells were subtracted from those in the positive wells. For graphical representations, negative responses were plotted with a value of 1 Spots Forming Unit (SFU)/10^6^ PBMCs.

### Flow cytometric analysis of RBD-specific memory B cells

Fluorescent SARS-CoV-2-specific RBD (SinoBiological) probes were prepared by combining biotinylated proteins with fluorescently labeled streptavidin. The RBD probes were prepared with a 4:1 molar ratio of RBD monomers to streptavidin, with one probe labeled with APC (BD) and the other labeled with PE (BD). This method was used in this assay to increase the detection specificity of SARS-CoV-2-specific B cells. The cells were first stained with a viability stain (BD) for 15 minutes at 4°C. The cells were subsequently washed with FBS and stained with a mixture of the three probes for 30 minutes at 4°C. After that, the cells were washed twice and then stained with anti-CD3-APC-Cy7 and anti-CD19-PE-Cy7 antibodies for 25 minutes at 4°C. The cells were washed twice and resuspended in 4% paraformaldehyde.

### *Ex vivo* activation-induced marker expression assay

First, 1x10^6^ cells in 200 mL of medium per well were seeded in a 96-well round-bottom plate and rested overnight in a humidified incubator at 37°C with 5% CO2. After 16 hours, the cells were stimulated for 24 hours with wild-type Spike peptide megapools at a final concentration of 0.25 μg/mL. Matched unstimulated samples from each donor at each time point were treated with costimulation alone. Twenty hours poststimulation, antibodies against CXCR5 and CD107a were added to the culture, and monensin (GolgiStop, BD Biosciences) was then added for staining for four hours at 37°C. After four hours, the cells were washed once with PBS supplemented with 2% FBS. The cells were stained for 15 minutes at room temperature with Live/Dead 510 and Fc receptor blocking solution (Human TruStain FcX, BioLegend) and washed once in FACS buffer. Surface staining was then performed for 20 minutes at room temperature with antibodies directed against CD3, CD4, CD8, CD69, CD137, PD-1, TIGIT, CD38, HLA-DR, and OX40 in FACS buffer. The cells were washed once in FACS buffer, fixed and permeabilized for 30 minutes at 4°C (eBioscience Foxp3/Transcription Factor Fixation/Permeabilization Concentrate and Diluent), and washed once in 1X permeabilization buffer prior to staining for intracellular IFN-γ and IL-21 at 4°C for one hour. The cells were then washed once and resuspended in 4% paraformaldehyde prior to data acquisition.

## Results

### Study design and characteristics of the enrolled PLWH and healthy controls

The participants, namely, fifty PLWH on ART and thirty HCs, were recruited between February 2021 and December 2022, as described in **Table 1**. To investigate the kinetics of immune responses following both primary and booster vaccine doses, we collected blood samples at seven different time points: pre-vaccine baseline (T0), one month after the first dose (T1), one month after the second dose (T2), the day of the third dose (T3), one month after the third dose (T4), three months after the third dose (T5) and twelve months after the third dose (T6). Owing to limitations of the study conditions, we could not collect blood samples from HCs immediately before the third dose. As shown in **Table 1**, the median age of the PLWH was 35.00 years, and 94% were male. The median age of the HCs was 39.00 years, and 80% were male. Those participants did not develop SARS-CoV-2 infection during the follow-up period. The demographic characteristics of the enrolled participants are provided in **Table 1**.

**Table 1:** Study design and characteristics of the subjects enrolled in our study.

	PLWH (n=50)	HCs (n=30)	*P* value
Male (%)	47/50 (94%)	24/30 (80%)	0.055
Age (years)	35.00 (25.00-41.25)	39.00 (35.00-48.50)	0.112
HBV infection (%)	0.00	0.00	
HCV infection (%)	1.00 (2%)	0.00	
CD4 count at T0 (cells/µL)	608.50 (513.75-734.75)	/	
CD4 count at T3 (cells/µL)	605.00 (510.50-772.75)	/	
CD4 count at T6 (cells/µL)	603.50 (497.50-804.75)		
CD4 count (cells/µL)			
<500, n (%)	13 (26%)	/	
>500, n (%)	37 (74%)	/	
VL (copies/mL)	TND	/	
Antiretroviral treatment			
INSTI +NRTI /2NRTIs	4 (8%)	/	
NNRTI+NRTI	4 (8%)	/	
Elvitegravir	6 (12%)	/	
NNRTI + 2NRTIs	31 (62%)	/	
Others	5 (10%)	/	
SARS-CoV-2 infection status			
Convalescent patients at study entry	0 (0%)	0 (0%)	
Patient post-vaccination	0 (0%)	0 (0%)	

HCs, healthy controls; PLWH, people living with HIV; VL, viral load.

### SARS-CoV-2 vaccination induces durable SARS-CoV-2-specific B-cell responses

It has previously been shown that infection and vaccination result in the production of SARS-CoV-2-specific MBCs in proportion to serological responses (27, 28). To explore whether long-lasting B-cell immune memory can be induced by SARS-CoV-2 vaccination, we assessed the frequency of RBD-reactive specific B-cells. In this study, owing to the limited number of samples, we measured the dynamic frequencies of SARS-CoV-2-specific B cells in PLWH and HCs on the day of the third vaccination (T3) and at three subsequent time points (T4, T5 and T6) after the third dose. In our study, SARS-CoV-2 spike-specific IgG and IgM antibodies were tested for their ability to bind to the SARS-CoV-2 RBD protein via ELISA, and NAb titers were measured against the ancestral vaccine-matched Wuhan Hu-1SARS-CoV-2 (WT) strain via pseudovirus neutralization at T3 and T4.

**Figure 1:**
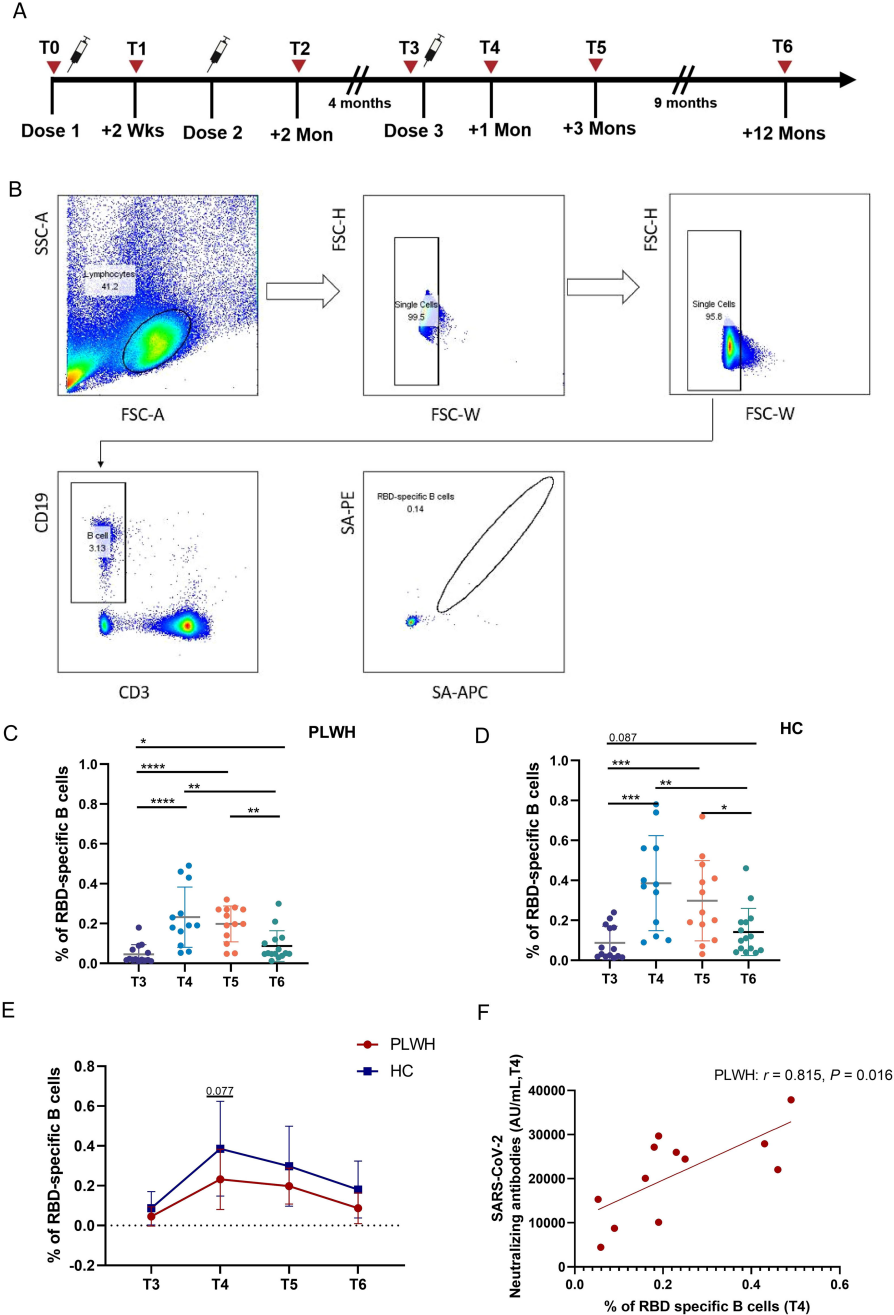
Vaccine-induced SARS-CoV-2-specific B-cell responses in PLWH and HCs after administration of the third dose. **(A)** Study design for our vaccine cohort. **(B)** Gating strategy used for flow cytometric analysis of SARS-CoV-2-RBD-specific B cells according to dual positivity for Spike-PE and Spike-APC to exclude nonspecific binding in subject post-vaccination. **(C, D)** Percentages of RBD-specific B cells after the booster dose of inactivated SARS-Cov-2 vaccine in PLWH and HCs. **(E)** Comparison of RBD-specific B-cell frequencies in PLWH and HCs at different time points after the booster vaccination. **(F)** Correlation between the percentage of RBD-specific B cells and the neutralizing antibody titer; statistical test: Spearman’s rank–order correlation. *P <0.05; **P <0.01; ***P <0.001; ****P <0.0001.

As described in a previous report (21), the humoral response was strongly enhanced after the third dose in PLWH and controls (**Supplementary Figure S1**). There were no differences in the SARS-CoV-2 RBD-specific IgG and IgM levels between the two groups at T3 or T4, while PLWH had lower NAb titers than HCs did at T3 (**Supplementary Figure S1**). In this study, we used a fluorescence-labeled ectodomain of the RBD protein to measure the frequency of SARS-CoV-2-specific B cells, and the study design for our vaccine cohort and the related flow cytometry gating strategy is shown in **Figures 1A, B**. **Figures 1C, D** show that the frequencies of SARS-CoV-2-specific B cells in PLWH and HCs were greater at T4, T5 and T6 than at T3, although the proportion of RBD-specific B cells in HCs at T6 was not significantly greater than that at T3 (*P*=0.087). Additionally, the frequency of SARS-CoV-2-specific B cells in both PLWH and HCs was significantly lower at T6 than at T4 and T5. As shown in **Figure 1E**, no significant difference was observed in the percentage of specific B cells between PLWH and HCs either before or after the third dose of inactivated vaccine, except at T4. At this time point, the percentage tended to be greater in PLWH than in HCs (*P* =0.077). Notably, in PLWH, a positive association was found between the titer of NAbs and the percentage of RBD-specific B cells at T4 (**Figure 1F**).

### Dynamic changes in the frequencies of bulk B cells, plasma cells and CD27^+^memory B cells after SARS-CoV-2 vaccination

**Figure 2:**
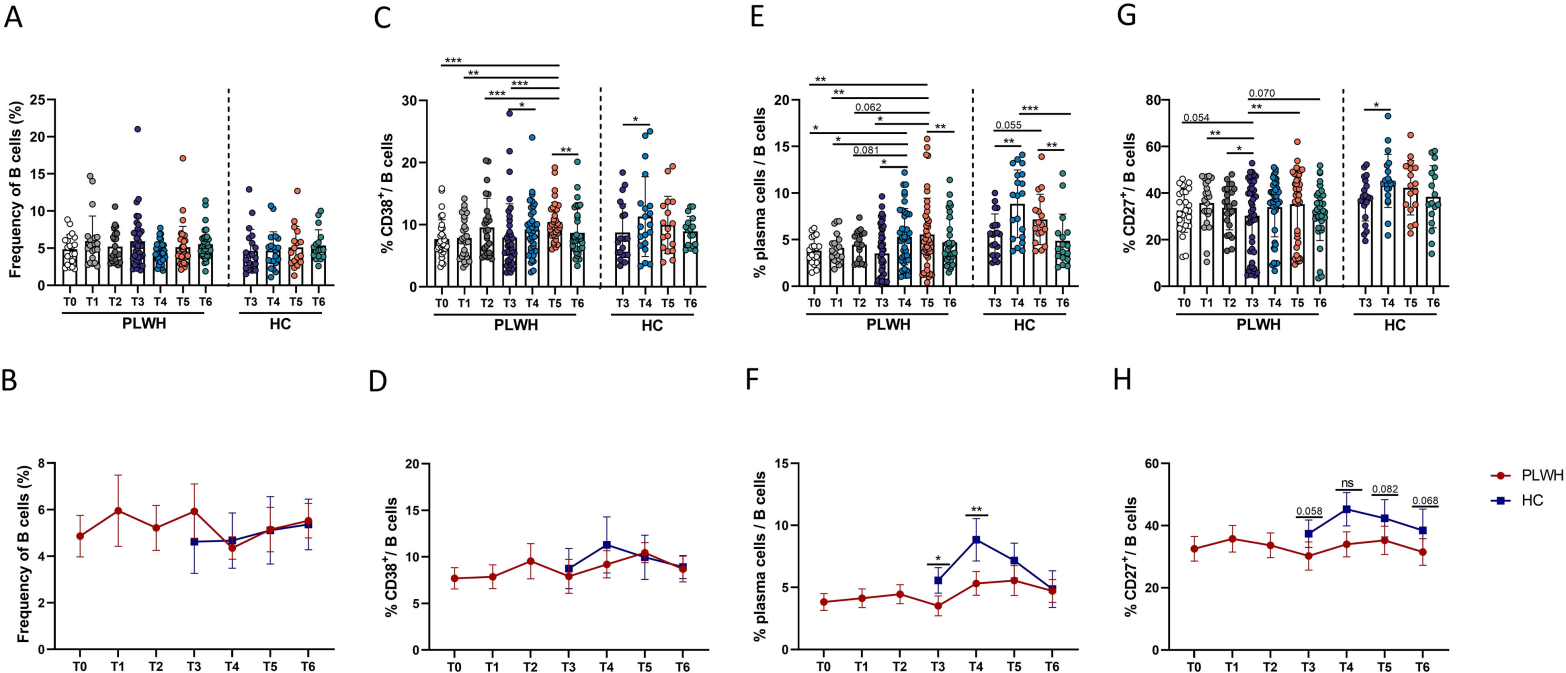
Dynamic changes in the frequencies of B cells and B-cell subsets following inoculation with inactivated SARS-CoV-2 vaccines in PLWH and HCs. **(A, B)** Percentages of CD3^-^CD19^+^ B cells in PLWH and HCs before and after administration of the SARS-CoV-2 vaccine. **(C, D)** Expression of CD38 on B cells in PLWH from T0 to T6 and in HCs from T3 to T6. **(E, F)** Frequencies of plasma cells (defined as CD27^+^IgD^-^CD38^+^ B cells) in PLWH after the first, second and third doses of the SARS-CoV-2 vaccine and in HCs after the third dose. **(G, H)** Proportions of CD27^+^ B cells following full vaccination course in PLWH and HCs (after the booster vaccination). *P <0.05; **P <0.01; ***P <0.001; ****P <0.0001. Red represents PLWH; blue represents HCs.

To increase our understanding of the complementary role of B-cell-mediated immune responses after vaccination, the characteristics of B cell populations, including the dynamic changes in the populations of B cells and B-cell subsets, such as plasma cells and CD27^+^ memory B cells, were evaluated. In contrast to the findings for SARS-CoV-2-RBD-specific B cells, there was no significant change in the percentage of bulk B cells after SARS-CoV-2 vaccination (**Figure 2A**). However, the frequency of CD38-expressing B cells in PLWH increased after vaccination, peaked at T5, and then decreased at T6. At this time point, the frequency of CD38^+^ B cells did not differ from that at T0 (**Figure 2C**). There were no differences in the proportions of bulk B cells and CD38^+^ B cells between PLWH and HCs (**Figures 2B, D**).

Given the important components of B-cell immune responses, the characteristics of plasma cell and CD27^+^ memory B cell populations were further evaluated. In this study, we measured the frequencies of plasma cells, which were defined as IgD^-^CD27^+^ CD38^+^ B cells. As shown in **Figure 2E**, we observed significant expansion of plasma cells, consistent with previous reports (29, 30). Although no significant change in the percentage of plasma cells was observed after the first- and second vaccine doses, the third dose (booster) led to significant expansion of plasma cells in PLWH. However, the frequency of plasma cells returned to pre-vaccination levels over time. Interestingly, the percentage of peripheral plasma cells in PLWH was significantly lower than that in HCs at both T3 and T4 (**Figure 2F**). Next, the impact of SARS-CoV-2 vaccination on CD27^+^ memory B cells was assessed. As shown in **Figure 2G**, population of CD27^+^ memory B cells, which was decreased in PLWH at T3, was found to undergo significant expansion after the third vaccine dose. In addition, the frequency of CD27^+^ memory B cells in PLWH was lower than that in HCs at T3 and at the three subsequent timepoints after the third vaccination, and the difference exhibited a trend toward statistical significance (**Figure 2H**).

In addition, the frequencies of plasma cells at T4 after the third dose in PLWH were found to be positively correlated with NAb titers (**Supplementary Figure S2**).

### Durable SARS-CoV-2-specific T-cell responses are elicited by inactivated SARS-CoV-2 vaccines

**Figure 3:**
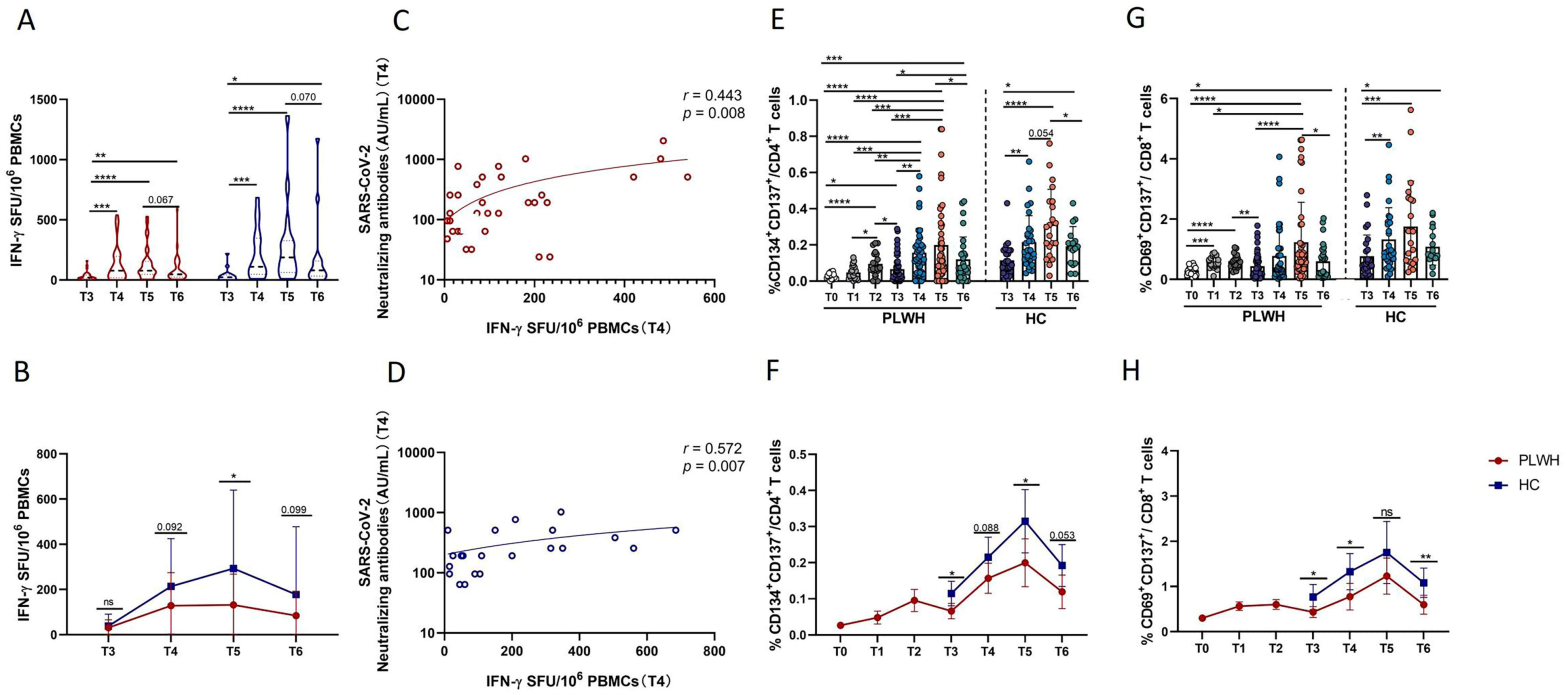
Evaluation of SARS-CoV-2-specific T-cell responses via IFN-γ ELISpot and activated induced marker (AIM) assays. **(A)** Grouped scatter plot comparing the IFN-γ ELISpot spot-forming units (SFUs) per 10^6^ peripheral blood mononuclear cells (PBMCs). PBMCs from HCs or PLWH after administration of the booster vaccination were stimulated with peptides covering the sequence of the wild-type spike protein. **(B)** Comparison of IFN-γ ELISpot spot-forming units (SFUs) per 10^6^ PBMCs between PLWH and HCs after the booster dose of the SARS-CoV-2 vaccine. **(C, D)** Correlations between specific T-cell responses (measured by IFN-γ release) and titers of neutralizing antibodies. **(E)** Frequencies of Spike-specific CD4^+^ T cells after the primary and booster vaccinations in PLWH and after the booster vaccination in HCs. **(F)** Dynamic changes in the frequencies of spike-specific CD4^+^ T cells in PLWH from T0 to T6 and in HCs from T3 to T6. **(G)** Proportions of Spike-specific CD8^+^ T cells in PLWH after the full course of vaccination and in HCs after the booster dose of the SARS-CoV-2vaccine. **(H)** Dynamic changes in the frequencies of spike-specific CD8^+^ T cells in PLWH from T0 to T6 and in HCs from T3 to T6. Red represents PLWH; blue represents HCs.

T-cell immunity is involved in adaptive immunity and is crucial for the defense against SARS-CoV-2 infection. First, in this study, the magnitude of SARS-CoV-2-spike-specific T-cell responses was assessed cross-sectionally *via* an IFN-γ ELISpot assay with overlapping peptide pools covering the complete sequence of the wild-type spike glycoprotein. The results of the ELISpot assay revealed that SARS-CoV-2-specific T-cell responses were induced in both PLWH and HCs after the third dose of inactivated vaccine (**Figure 3A**). Notably, in both PLWH and HCs, the magnitude of T-cell responses at T4 was significantly greater than that at T3 and remained relatively stable at T5. Moreover, an obvious decrease in the magnitude of T-cell responses was observed at T6 compared with T5, but it was still stronger than that at the time of the third vaccine dose (**Figure 3A**). In addition, compared with HCs, PLWH showed a weaker T-cell response, especially at T5 (**Figure 3B**). Additionally, we further assessed the relationship between T-cell responses and humoral responses induced by inactivated SARS-CoV-2 vaccine. As shown in **Figures 3C, D**, the strength of T-cell responses measured by ELISpot was positively associated with the titer of SARS-CoV-2 NAbs in both PLWH and HCs at T4.

Next, the full breadth of the SARS-CoV-2-specific T-cell response was analyzed via *ex vivo* stimulation with SARS-CoV-2 spike peptide pools and subsequent flow cytometric analysis. SARS-CoV-2-specific CD4^+^ T cells were defined as CD4^+^ T cells with coexpression of CD134 and CD137, while CD8^+^ T cells with coexpression of CD69 and CD137 were defined as SARS-CoV-2-specific CD8^+^ T cells. As shown in **Figures 3E, G**, robust expansion of SARS-CoV-2-specific CD4^+^ T cells was observed in our study. Spike-specific CD4^+^ T and CD8^+^ T cells displayed similar kinetics; specifically, in PLWH, the frequencies of spike-specific CD4^+^ and CD8^+^ T cells were significantly greater after primary vaccination than at T0, and more distinct increases in these two spike-specific subsets of cells were observed following the third vaccination. Notably, at T6, SARS-CoV-2-specific CD4^+^ and CD8^+^ T-cell responses were still stronger than those at T0 and even at T3 in PLWH (**Figures 3E, G**). Interestingly, at T3 and T5, PLWH had significantly weaker CD4 T-cell responses than HCs did at the same time point (**Figure 3D**). Moreover, although there was no difference at T5 in terms of the magnitude of specific CD8^+^ T-cell responses, PLWH had lower frequencies of SARS-CoV-2-specific CD8^+^ T cells at T3, T4 and T6 than HCs did (**Figure 3H**). Taken together, these findings suggest that the full breadth of SARS-CoV-2-specific CD4^+^ and CD8^+^ T-cell responses can be induced and maintained for at least one year after the third SARS-CoV-2 vaccination. In addition, PLWH exhibited weaker peak SARS-CoV-2-specific CD4^+^ and CD8^+^ T-cell responses than HIV-uninfected individuals did.

### Dynamic changes in the frequencies of activated, exhausted and SARS-CoV-2-specific functional CD4 T cells following SARS-CoV-2 vaccination

**Figure 4:**
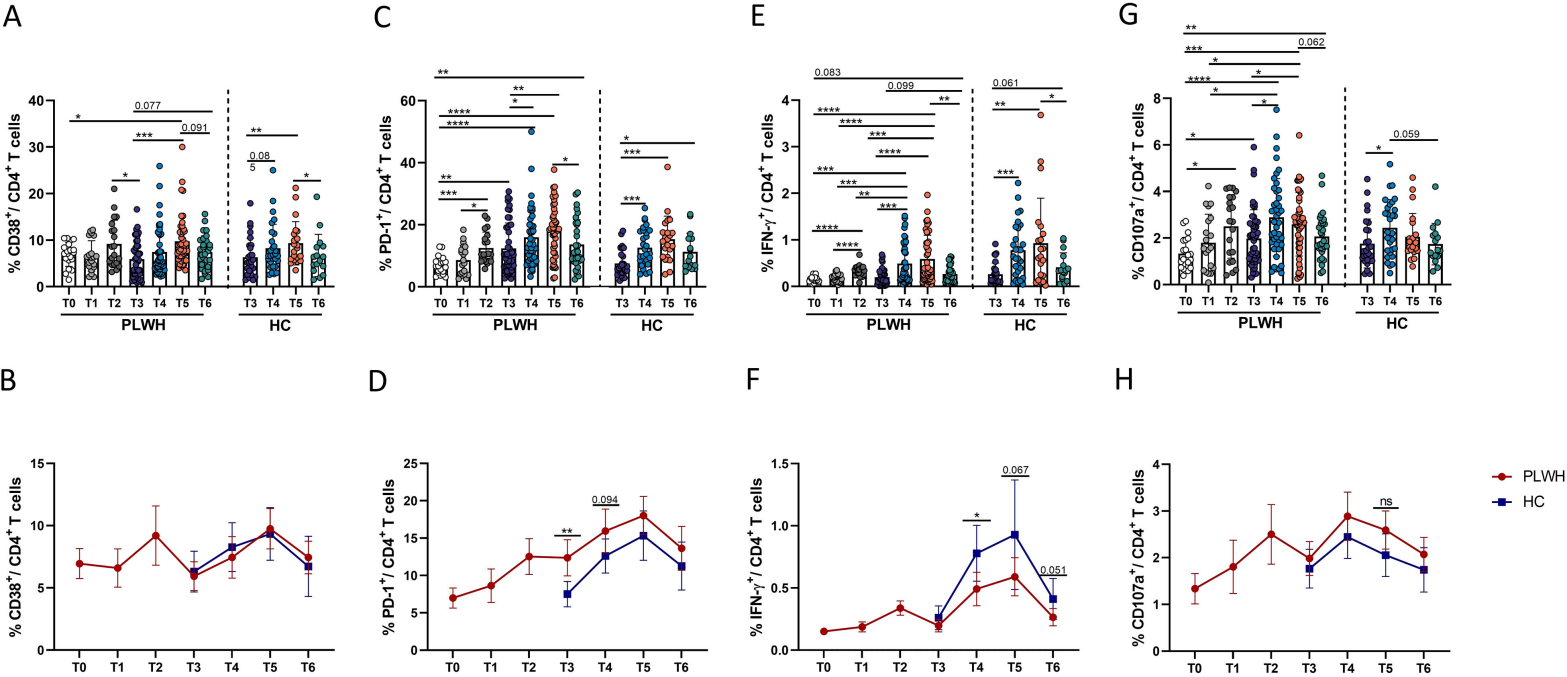
Dynamic phenotypic and functional changes in CD4^+^ T cells in PLWH and HCs after SARS-CoV-2 vaccination. **(A, B)** Activation of CD4^+^ T cells was identified by the expression of CD38. The line chart in **(B)** shows the dynamic changes in CD4^+^ T-cell activation over time after SARS-CoV-2 vaccination in PLWH and HCs. **(C, D)** Exhaustion of CD4^+^ T cells was identified by the expression of PD-1. The line chart in **(D)** shows the dynamic changes in CD4^+^ T-cell exhaustion over time after SARS-CoV-2 vaccination in PLWH and HCs. **(E, F)** Expression of IFN-γ in CD4^+^ T cells stimulated with spike peptide pools in PLWH from T0 to T6 and in HCs from T3 to T6. **(G, H)** Expression of CD107a in CD4^+^ T cells stimulated with spike peptide pools in PLWH from T0 to T6 and in HCs from T3 to T6. Red represents PLWH; blue represents HCs.

To increase our understanding of the complementary role of cellular immunity after vaccination, we measured the changes in the populations of activated, exhausted and functional CD4^+^ T cells. As shown in **Figures 4A-D**, CD4^+^ T-cell activation and exhaustion in PLWH were significantly greater after the primary and booster doses of the SARS-CoV-2 vaccine than at T0. At T6, the frequency of activated CD4^+^ T cells was comparable to that at T0, while the frequency of PD-1^+^ CD4^+^ T cells was greater than that at T0 (**Figures 4A, C**). There was no obvious difference in the frequency of CD38^+^ CD4^+^ T cells between PLWH and HCs; however, PLWH had more PD-1^+^ CD4^+^ T cells than HCs did after the third dose of activated SARS-CoV-2 vaccine (**Figure 4D**).

Next, the ability of CD107a and IFN-γ to be secreted by CD4^+^ T cells was further analyzed to determine the characteristics of CD4^+^ T-cell functional activity in this cohort. As shown in **Figures 4E-H**, the data revealed that the frequency of functional CD4^+^ T cells was significantly increased after administration of inactivated SARS-CoV-2 vaccine, especially the third dose. However, unlike the patterns observed for the activation and exhaustion of CD4^+^ T cells mentioned above, the frequencies of functional CD4^+^ T cells, such as CD107a-releasing CD4 T cells, were still significantly greater at T6 than at T0 in PLWH (**Figure 4G**). Moreover, there were fewer functional CD4^+^ T cells in HIV-infected individuals than in HCs, as shown in **Figure 4F**.

### cTfh cell frequencies are increased after SARS-CoV-2 vaccination and are related to the magnitude of the humoral neutralizing antibody response

**Figure 5:**
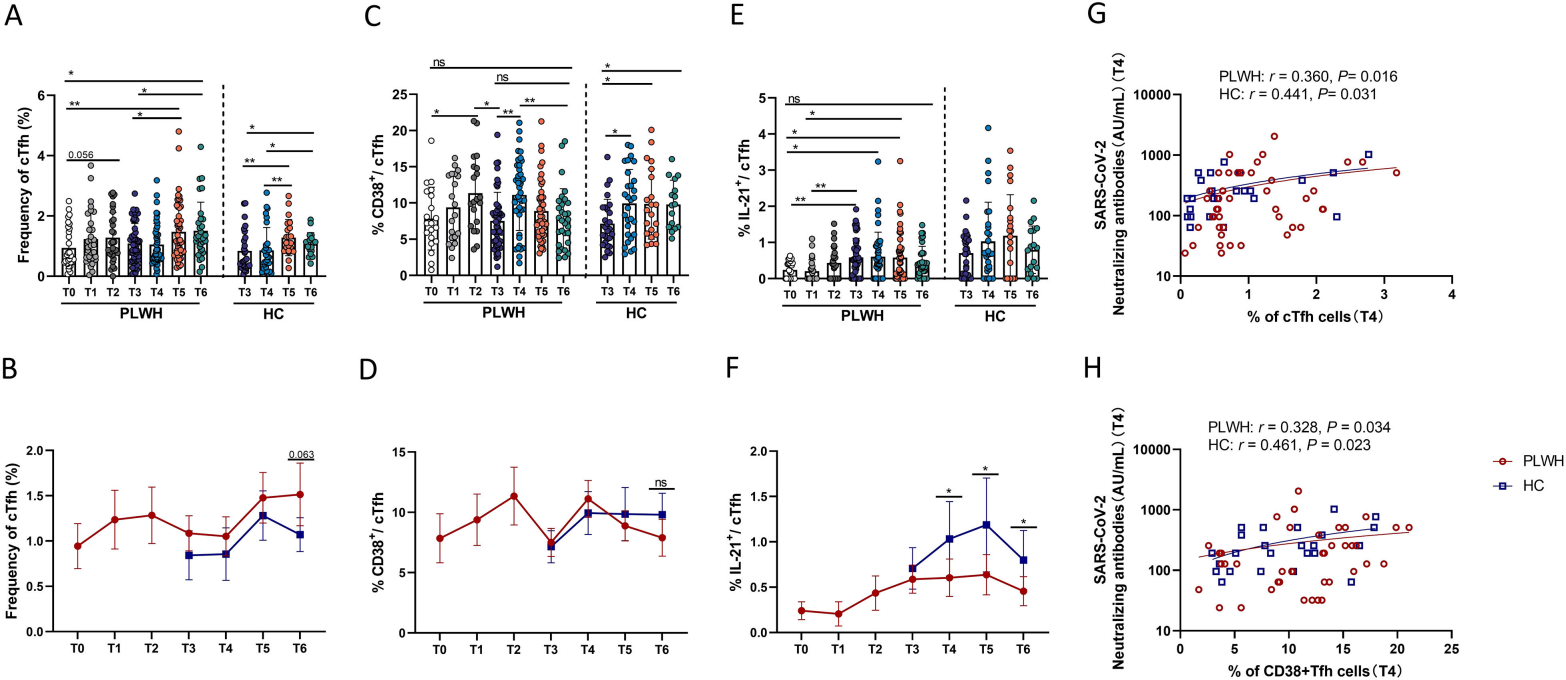
The impact of SARS-CoV-2 vaccination on cTfh cells and cTfh cell subsets. **(A, B)** Percentages of cTfh cells in PLWH from T0 to T6 and in HCs from T3 to T6. **(C, D)** Percentages of CD38^+^ cTfh cells in PLWH from T0 to T6 and in HCs from T3 to T6. **(E, F)** Percentages of IL-21^+^ cTfh cells in PLWH from T0 to T6 and in HCs from T3 to T6. **(G)** Correlations between the frequencies of cTfh cells and neutralizing antibody titers at T4 in PLWH and HCs. **(H)** Correlations between the frequencies of CD38^+^ cTfh cells and neutralizing antibody titers at T4 in PLWH and HCs.

The frequencies of circulating Tfh (cTfh) cells (CXCR5^+^ PD-1^+^ CD4^+^ T cells) changed with the administration of vaccine doses and the time interval between vaccinations. Expansion of cTfh cells was not observed at T1, but slight expansion was observed at T2, and the third dose of SARS-CoV-2 vaccine led to significant expansion of cTfh cells at T5 and T6 in PLWH (**Figure 5A**). Similarly, the population of cTfh cells was significantly increased at T5 and T6 in HCs after inoculation with the third vaccine dose (**Figure 5A**). In addition, as shown in **Figure 5B**, there was no significant difference in the percentage of cTfh cells between PLWH and HCs, except that at T6, PLWH had more cTfh cells than HCs did, with a trend toward a statistically significant difference (*P*=0.063). Moreover, as shown in **Figure 5C**, we noted that the frequency of activated cTfh cells (defined as CD38^+^ cTfh cells) in PLWH was significantly increased at T2 after the second vaccination compared with T0 and was decreased at T3 compared with T2. At T4, one month after the third vaccination, the frequency of activated cTfh cells was increased compared with that at T3 after which the frequency of these cells decreased over time. The percentage of CD38^+^ cTfh cells at T6 was comparable to that at T3 in PLWH, whereas in HCs, these cells expanded after the third vaccination and were still more abundant at T6 than at T3. There was no difference in the percentage of activated cTfh cells between PLWH and HCs (**Figure 5D**). Moreover, the ability of cTfh cells to release IL-21 was assessed in this study. In line with the findings for cTfh and CD38^+^ cTfh cells, the population of IL-21-releasing cTfh cells in PLWH was increased after vaccination. However, PLWH still had a lower proportion of IL-21-releasing cTfh cells than HCs did, although the proportion of these cells in HCs did not increase significantly after the third vaccination (**Figures 5E, F**). In addition, as shown in **Figures 5G, H**, correlation analysis revealed that the percentages of cTfh and CD38^+^ cTfh cells were positively associated with the titer of neutralizing antibodies, suggesting that cTfh cells play a role in mediating the interaction between humoral and adaptive T-cell responses.

### Dynamic changes in the frequencies of activated, exhausted and SARS-CoV-2-specific functional CD8^+^ T cells following SARS-CoV-2 vaccination

**Figure 6:**
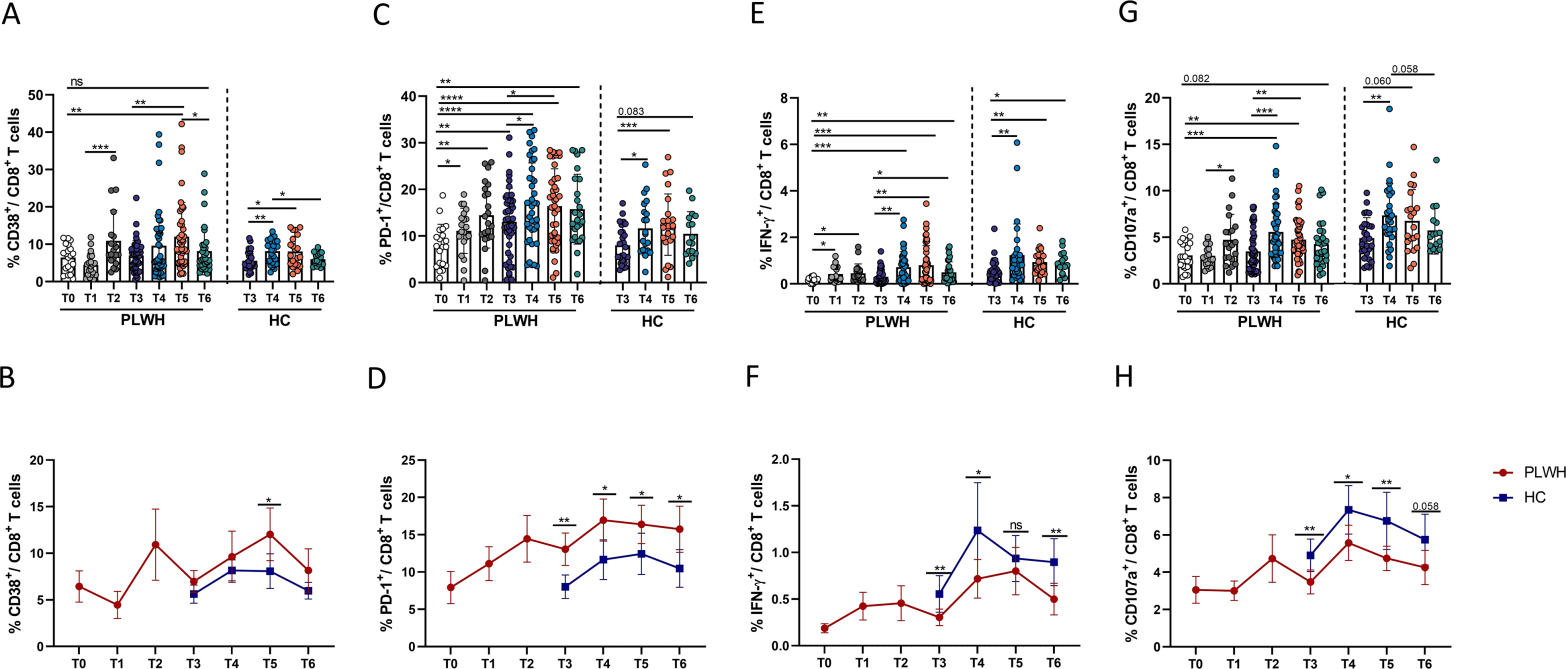
Dynamic phenotypic and functional changes in CD8^+^ T cells in PLWH and HCs after SARS-CoV-2 vaccination. **(A, B)** Dynamic changes in activated CD8^+^ T cells (CD38^+^CD8^+^ cells) over time in PLWH and HCs after SARS-CoV-2 vaccination. **(C, D)** Dynamic changes in exhausted CD8^+^ T cells (PD-1^+^CD8^+^ cells) in PLWH and HCs over time after SARS-CoV-2 vaccination. **(E, F)** Dynamic changes in the production of IFN-γ-releasing CD8^+^ T cells in PLWH and HCs over time after SARS-CoV-2 vaccination. **(G, H)** Dynamic changes in the production of CD107a-releasing CD8^+^ T cells in PLWH and HCs over time after SARS-CoV-2 vaccination. Red represents PLWH; blue represents HCs.

Next, we measured the activation, exhaustion and changes in the function of CD8^+^ T cells. Generally, the frequencies of CD8^+^ T cells exhibited dynamic changes similar to those observed for CD4^+^ T cells in both PLWH and HCs. Although activated CD8^+^ T cells (defined as CD38^+^ CD8^+^ T cells) in PLWH and HCs underwent expansion at some time after vaccination, the frequency of activated CD8^+^ T cells in both groups decreased and returned to the level observed at the time of the third vaccination (**Figures 6A, B**). However, one year after the third dose, there were still more exhausted CD8^+^ T cells (PD-1^+^ CD8^+^ T cells) in both PLWH and HCs than at the time of the third vaccine dose (**Figures 6C, D**). Moreover, the numbers of activated and exhausted CD8^+^ T cells in PLWH were significantly greater than those in HCs at all time points after the third vaccination (**Figures 6B, D**). To determine the changes in SARS-CoV-2-specific CD8^+^ T-cell functions, we detected the expression of CD107a and IFN-γ on CD8^+^ T cells. As shown in **Figures 6E, G**, the percentage of functional CD8^+^ T cells was significantly increased after SARS-CoV-2 vaccination, especially after the third dose. As shown in **Figures 6F, H**, PLWH had fewer functional CD8^+^ T cells than HCs did.

## Discussion

Establishing immune memory is crucial in the defense against SARS-CoV-2 infection; thus, it is essential to know how long the immune response to SARS-CoV-2 will persist after SARS-CoV-2 vaccination, especially in PLWH with impaired immunity. In this study, we investigated the B- and T-cell responses induced by inactivated SARS-CoV-2 vaccine in a longitudinal cohort of HIV-infected individuals to determine the vaccine efficiency and the maintenance time of the immune response. Our data demonstrated that robust SARS-CoV-2-specific B-cell and T-cell responses were induced after the primary dose and booster doses of inactivated SARS-CoV-2 vaccine in PLWH who are well controlled on ART and was sustained for at least one year after the booster vaccination.

Previous studies have shown that the antibody responses during the SARS-CoV-2 infection recovery period are further substantiated by the continued increases in both the spike and RBD memory B-cell responses for more than 3–5 months before they plateau over 6–8 months (11, 31, 32). In addition, several studies have shown that the frequencies of SARS-CoV-2-specific CD4^+^ and CD8^+^ memory T cells peak early, within the first month, but then slowly decline over the next 6–7 months in convalescent patients infected with SARS-CoV-2 (18). The production of antigen-specific memory B and T cells was induced by booster vaccination and lasted for at least 6 months (10). In our study, RBD-specific B cells and spike-specific T cells were detected one year after the booster shot in PLWH and controls, which suggests that lasting immune memory could be induced by inactivated SARS-CoV-2 vaccine. However, the percentage of RBD-specific B cells (memory B cells, plasma cells) and the magnitude of T-cell responses were lower than those in the control group. Considering that higher frequencies of RBD-specific memory B cells are associated with higher titers of neutralizing antibodies, the lower frequency of RBD-specific B cells in PLWH could contribute to the weaker humoral responses in these individuals than in HCs. Plasma cells are specialized antibody-producing cells that synthesize and secrete specific antibodies (33). In our study, plasma cells were found to undergo expansion after SARS-CoV-2 vaccination, and the finding that the frequency of plasma cells was positively correlated with the titer of neutralizing antibodies might confirm the prompt mobilization of B cells, guaranteeing an early humoral response in PLWH. In addition, the percentage of plasma cells in PLWH at T6 was not different from that at T0, indicating that humoral immunity may not be sufficient to prolong SARS-CoV-2 immunity. Besides, we further analyzed the relationship between humoral immunity and T-cell responses in both group subjects, and we found that the titer of SARS-CoV-2 NAbs was positive associated with the magnitude of T-cell responses measured by the IFN-γ ELISpot assay at T4 (**Supplementary Figure S3**).

We previously revealed that the proportion of T-cell responders 2 weeks after the administration of inactivated SARS-CoV-2 vaccines (first dose and second dose) was significantly lower among PLWH than among HIV-uninfected individuals, but the proportion was comparable in both groups of participants 1–2 months after the second vaccine dose (26). In this study, we observed that the magnitude of T-cell responses determined by ELISpot assays was greatly increased after the booster vaccinations compared with the primary vaccination in PLWH and peaked at 3 months following the third vaccination. Additionally, via AIM assays, our present study provides information on the full breadth of SARS-CoV-2-specific CD4^+^ and CD8^+^ T-cell responses in HIV-infected subjects and suggests that the third dose of vaccine has a significant effect on the magnitude of T-cell responses, consistent with our previous findings in HIV-uninfected participants after the third dose of inactivated SARS-CoV-2 vaccine (34). However, in contrast to the results of our study, T-cell immunity following three SARS-CoV-2 vaccine doses in PLWH was previously reported to be equal to that in uninfected controls (35). The frequencies of Spike-specific CD4^+^ T cells plateaued after the 2^nd^ dose, with no significant differences in the polyfunctional SARS-CoV-2-specific T-cell proportions between PLWH and uninfected controls after the 3^rd^ dose (35). These findings could be attributed to the fact that the subjects received different types of SARS-CoV-2 vaccines, such as mRNA and activated vaccines, in these studies.

Additionally, Hung et al. reported that the third shot boosted SARS-CoV-2 immunity, with better antibody responses and more robust IFN-γ and IL-2 responses of CD4^+^ and CD8^+^ T cells in PLWH than in individuals without HIV (36). A possible explanation is that the slightly compromised immunity in PLWH indeed preserved the functional capacity for a further response to the third shot or natural infection (36). Unfortunately, in this study, owing to the limited sample size, we did not have the ability to compare the differences in vaccine-induced immune responses between the initial dose and subsequent booster doses in the control group. In summary, in contrast to the humoral response reported in several studies (21, 37), our cohort displayed a stable cellular response throughout the one-year follow-up after the third vaccination. Moreover, we found that PLWH have impaired cellular immunity due to the lower peak T-cell responses measured by ELISpot assays and the lower percentage of spike-specific T cells shown by AIM assays, suggesting that cellular immune responses might be impaired by HIV infection even though PLWH have a relatively good immune status after effective ART, further emphasizing the importance of additional SARS-CoV-2 vaccinations for PLWH.

In this study, we found that the frequency of SARS-CoV-2-specific CD8^+^ T cells releasing IFN-γ peaked three months after the third vaccination in HIV-infected patients but one month after the third vaccination in HCs, indicating a delay in the peak CD8^+^ T-cell response due to HIV infection. Moreover, at different time points at the time of and after the third vaccination, the number of functional CD8^+^ T cells producing IFN-γ and CD107a in HCs was universally greater, albeit to varying degrees, than that in PLWH, confirming that the magnitude of CD8^+^ T-cell responses to the vaccine was decreased in PLWH. In addition, the frequency of CD69^+^ CD137^+^ CD8^+^ T cells in HCs at T6 was still greater than that at T3, whereas the percentage of these specific CD8^+^ T cells at T6 in PLWH was lower than that in HCs and comparable to that at T3, suggesting that PLWH may have shorter-lasting durability of specific full-breadth CD8^+^ T-cell responses. However, this phenomenon was not observed for CD134^+^ CD137^+^ CD4^+^ T cells, as the full-breadth specific CD4^+^ T-cell response at T6 was greater than that at T3 in both PLWH and HCs. However, the function of IFN-γ-releasing CD8 T cells in PLWH one year after the third vaccine dose was reduced but was still stronger than that at the time of the primary vaccination, while CD4^+^ T cells, this function was decreased at T6 to the level observed at T0.

Even after effective ART, PLWH generally display increased immune activation, which may be caused by multiple factors, including microbial translocation, residual low-level viral replication and coinfections (38–40). Therefore, the immune response to novel antigens is generally weaker than that in uninfected individuals (41). Our recent study revealed that PLWH have increased expression of activation and exhaustion markers on both CD4^+^ and CD8^+^ T cells and that the degrees of CD8^+^ T-cell activation and exhaustion were negatively correlated with T-cell responses in PLWH at one month and three months following the third dose of activated SARS-CoV-2 vaccine (42), which highlights the impact of CD8^+^ T-cell activation and exhaustion on T-cell responses and possibly explains why PLWH mount weaker T-cell responses to SARS-CoV-2 vaccines. In this study, even at T6, PLWH still showed higher expression of PD-1 on CD8^+^ T cells, suggesting that the strength and persistence of T-cell responses are affected by HIV infection.

IL-21 is involved in T-cell-dependent B-cell activation in germinal centers, which enables the rapid proliferation of B cells and their subsequent differentiation into memory and long-lived plasma cells (43). A study of the mRNA-1273 vaccine-induced SARS-CoV-2-specific memory T-cell response revealed an increase in IL-21 expression in memory T cells in patients with kidney disease (44). An interesting finding of our study was the association between neutralizing antibody titers and the frequency of IL-21^+^CD4^+^ T cells in both PLWH and HCs (**Supplementary Figure S4**). These data highlight the critical role of CD4^+^ T cells that secrete IL-21 in the production of antibodies after the administration of SARS-CoV-2 vaccines. Considering the important role of cTfh cells in mediating antibody production and that PLWH have a relatively lower percentage of IL-21^+^ cTfh cells than HCs do, we speculated that a lack of IL-21^+^ cTfh cells may have implications for attenuation of the humoral response. cTfh cells are considered important components of long-lasting immunity and the production of protective antibodies, and previous studies have shown that both SARS-CoV-2 infection and vaccination can trigger cTfh cell responses, accompanied by an increase in antibody titers (29, 30). Humoral responses are related to the magnitude of T-cell responses, and our findings corroborate the importance of cTfh cells in supporting effective B-cell responses after vaccination, since positive relationships were observed between the percentage of cTfh cells and activated cTfh cells and the titer of neutralizing antibodies (**Figures 5G-H**) and because the frequencies of cTfh cells and activated cTfh cells were increased after the third immunization. Overall, we verified the interaction between B cells and T cells following the administration of inactivated vaccine.

Our study has several limitations. First, owing to the limited number of samples, immune responses in HCs were assessed only after the booster dose of the SARS-CoV-2 vaccine. Second, in this study, we focused on changes in the bulk CD4^+^ T and CD8^+^ T-cell phenotype and function, and studies related to the activation, exhaustion and cytotoxic activity of SARS-CoV-2-specific CD4^+^ and CD8^+^ T cells were not performed. Furthermore, the HIV-positive participants enrolled in this study were those with a good immune status, and the immune responses of individuals who have a CD4^+^ T-cell count of less than 200 cells/μL and are not receiving ART need to be explored in the future. Finally, we studied the immune responses of T/B cells only in the peripheral blood, and the immune responses in the lymph nodes, gut-associated lymphoid tissue (GALT) and germinal centers in PLWH still need to be further studied and discussed.

## Conclusion

Taken together, our data provide a functional and phenotypic map of SARS-CoV-2-specific B-cell and T-cell immunity induced by inactivated SARS-CoV-2 vaccine in PLWH and suggest that specific immune memory could last at least one year after additional vaccine doses. Moreover, our study highlights the crucial role of SARS-CoV-2-specific CD8^+^ T cells in eliciting potent and durable immune responses in PLWH. The observation that the PLWH in this study had highly durable and functionally replete memory B- and T-cell responses further demonstrates the benefits of a prime–boost strategy to amplify coordinated vaccine-induced immune responses, which could benefit vulnerable persons.

## Data Availability

The original contributions presented in the study are included in the article/**Supplementary Material**. Further inquiries can be directed to the corresponding authors.
