# Supplementary material for: Dynamic SARS-CoV-2-specific B-cell and T-cell responses induced in people living with HIV after a full course of inactivated SARS-CoV-2 vaccine
Source: Front Immunol. 2025 Feb 25;16:1554409. doi: 10.3389/fimmu.2025.1554409 (PMC11893571; doi:10.3389/fimmu.2025.1554409)

**Supplementary Figure S1. Titers of SARS-CoV-2-specific antibodies produced in response to inactivated SARS-CoV-2 vaccine in PLWH and HCs.**

(A-C) SARS-CoV-2-RBD IgG (A), neutralizing antibodies (B), and RBD IgM (C) at T3 and T4 in PLWH and HCs.

(D-E) Correlations of SARS-CoV-2 RBD IgG titers with neutralizing antibody titers (D) and RBD IgM titers (E) in the two groups.

**
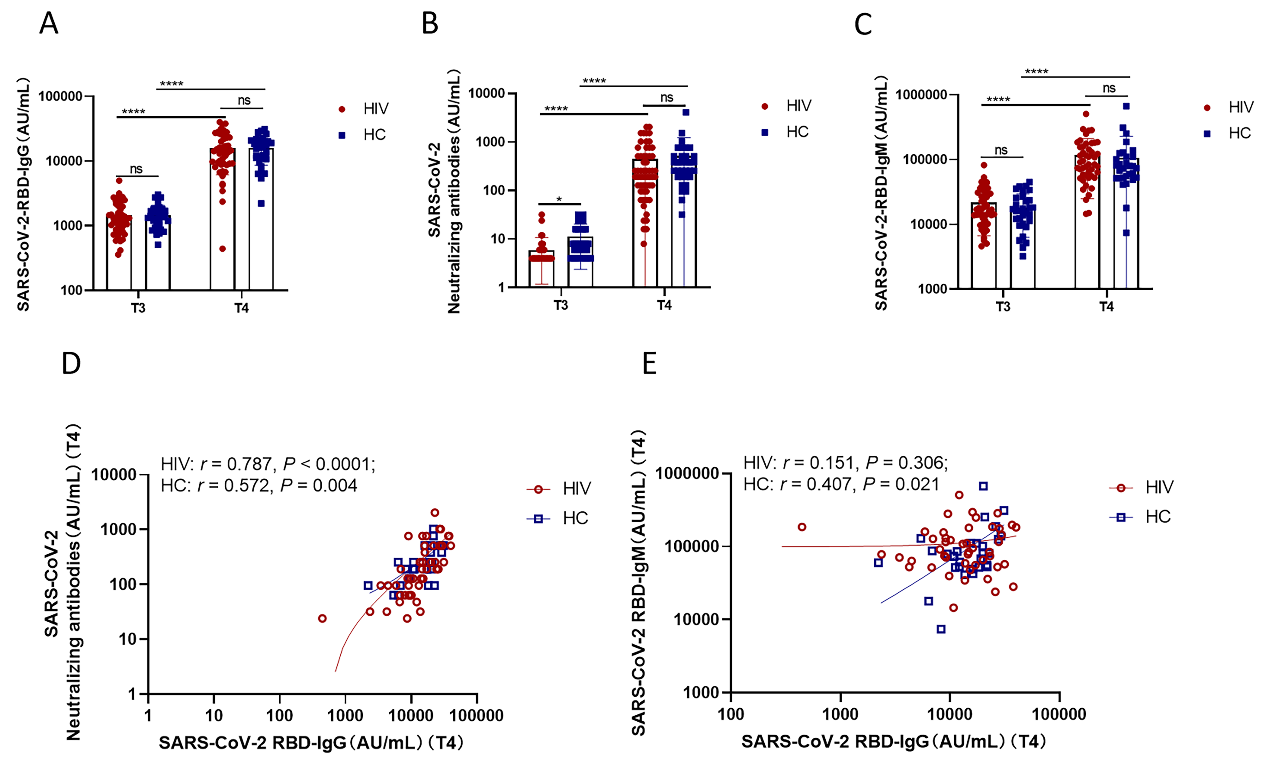
**

**Supplementary Figure S2. Correlation between the titer of SARS-CoV-2 neutralizing antibodies and the frequency of plasma cells at T4 in HIV-infected individuals.**


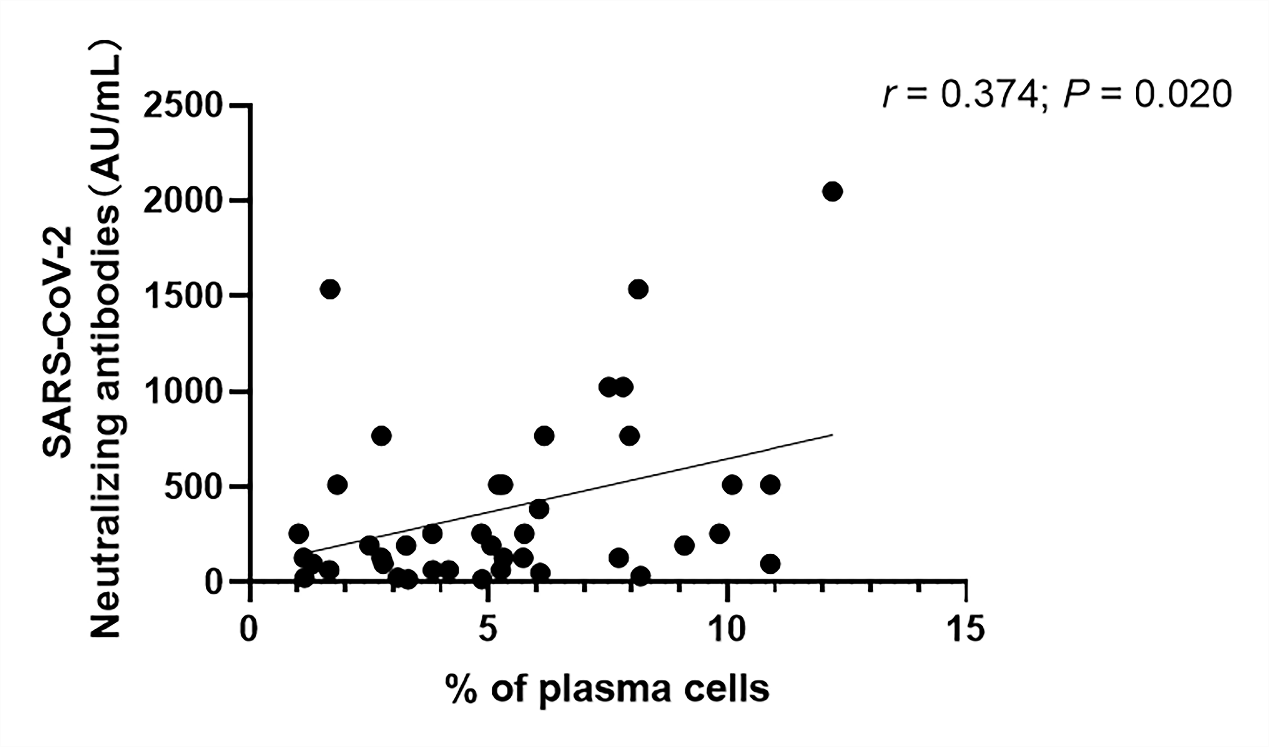


**Supplementary Figure S3. Correlation between the titer of SARS-CoV-2 neutralizing antibodies and the magnitude of T-cell responses measured by the IFN-γ ELISpot assay at T4 in HIV-infected individuals (A) and HCs (B).**

**
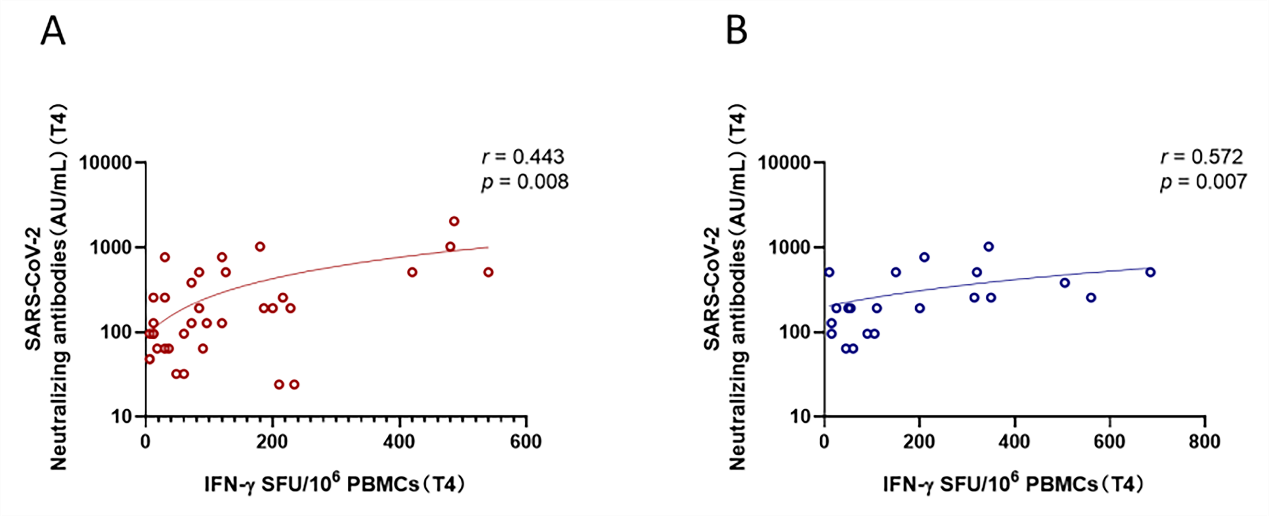
**

**Supplementary Figure S4. Correlation between the titer of SARS-CoV-2 neutralizing antibodies and the frequency of IL-21^+^CD4^+^ T cells at T4 in PLWH (A) and HCs (B).**


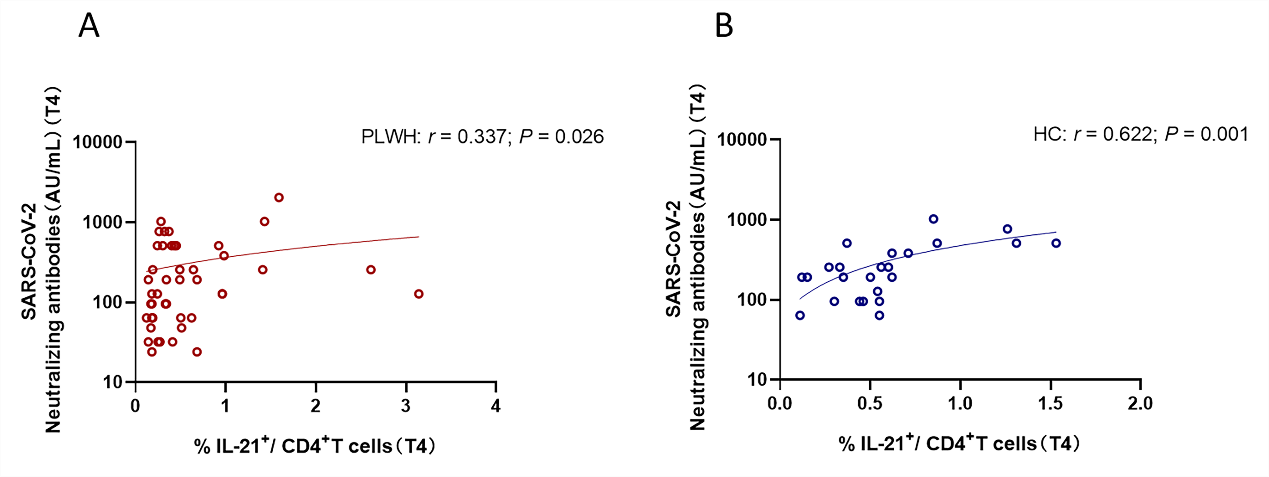

Supplement: Supplementary file 1 [file DataSheet1.docx]
